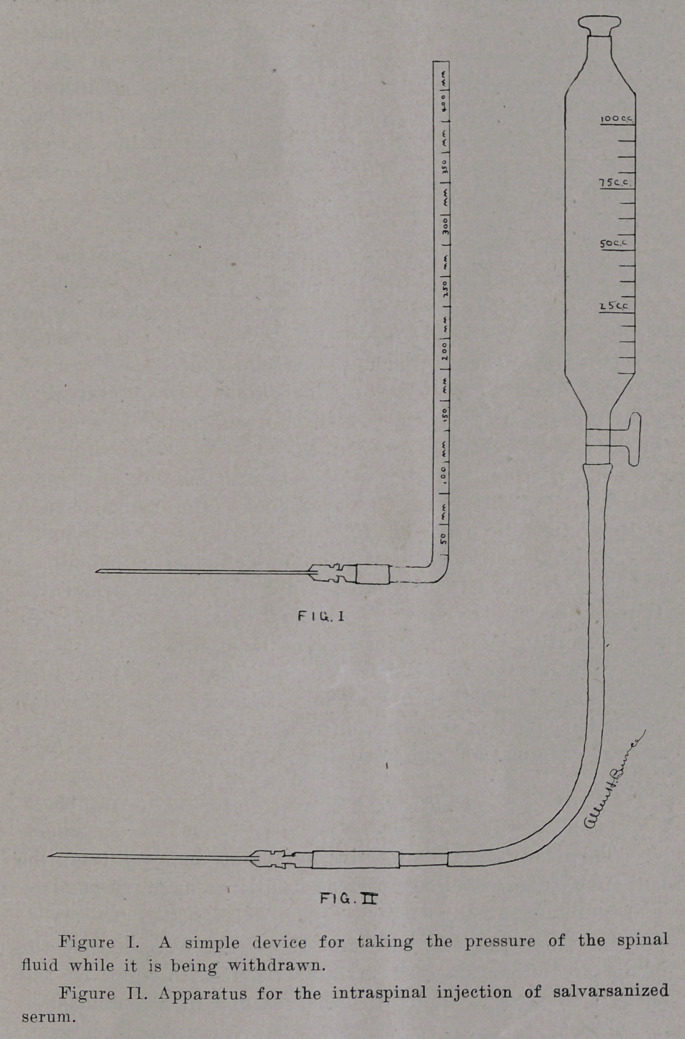# The Treatment of Nervous Diseases Following Syphilis

**Published:** 1914-07

**Authors:** Lewis M. Gaines, Allen H. Bunce

**Affiliations:** Atlanta, Ga.; Professor of Clinical Neurology, Atlanta Medical College; Atlanta, Ga.; Director of the Clinical Laboratory, Atlanta Medical College


### Journal-Record of Medicine

Successor to Atlanta Medical and Surgical Journal, Established 1855
and Southern Medical Record, Established 1870

**OWNED BY THE ATLANTA MEDICAL JOURNAL COMPANY**

PUBLISHED MONTHLY

*Official Organ Fulton County Medical Society, State Examining Board, Presbyterian Hospital, Atlanta, Birmingham and Atlantic Railroad Surgeons' Association, Chattahoochee Valley Medical and Surgical Association, Etc.*

EDGAR BALLENGER, M. D., Editor
BERNARD WOLFF, M. D., Supervising Editor
A. W. STIRLING, M. D., C. M., D. P. H.; J. S. HURT, B. Ph., M.D.
GEO. M. NILES, M. D., W. J. LOVE, M. D., (Ala.) ; Associate Editors
R. R. DALY, M. D., Assiciate Editor
E. W. ALLEN, Business Manager

**COLLABORATORS**

DR. W. F. WESTMORLAND, General Surgery
F. W. McRAE, M. D., Abdominal Surgery
H. F. HARRIS, M. D., Pathology and Bacteriology
E. B. BLOCK, M. D., Diseases of the Nervous System
MICHAEL HOKE, M. D., Orthopedic Surgery
CYRUS W. STRICKLER, M. D., Legal Medicine and Medical Legislation
E. C. DAVIS, A. B., M. D., Obstetrics
E. G. JONES, A. B., M. D., Gynecology
R. T. DORSEY, Jr., B. S., M. D., Medicine
L. M. GAINES, A. B., M. D., Internal Medicine
GEO. C. MIZELL, M. D., Diseases of the Stomach and Intestines
L. B. CLARKE, M. D., Pediatrics
EDGAR PAULIN, M. D., Opsonic Medicine
THEODORE TOEPEL, M. D., Mechano Therapy
A. W. STIRLING, M. D., Etc., Diseases of the Eye, Ear, Nose and Throat
BERNARD WOLFF, M. D., Diseases of the Skin
E. G. BALLENGER, M. D., Diseases of the Genito-Urinary Organs

VOL. LXI ATLANTA, GA., JULY, 1914. NO. 4

### THE TREATMENT OF NERVOUS DISEASES FOLLOWING SYPHILIS.

By LEWIS M. GAINES, M. D., Professor of Clinical Neurology, Atlanta Medical College, and ALLEN H. BUNCE, M. D., Director of the Clinical Laboratory, Atlanta Medical College.

Since the introduction of the Wassermann Reaction and the detailed study of the spinal fluid obtained by lumbar puncture, a surprisingly large number of nervous cases have been found to be of syphilitic origin.

These cases, formerly designated by a variety of names, whose etiology was obscure, may now be readily classified as syphilitic nervous diseases. Many of these conditions, especially if diagnosed early, will respond in a most gratifying manner to anti-syphilitic treatment. Tabes and paresis are included as forms of active syphilis of the nervous system. These conditions were formerly termed parasyphilis, but as Swift and Ellis remark, the term "parasyphilis" has lost its significance, and only the convenience its use possesses in classification justifies the retention of the expression. It must be said at once that paresis has not responded in a gratifying way to any method of treatment yet devised. The other forms of syphilis of the nervous system, however, including tabes, have yielded in a remarkable way to energetic treatment where the inflammatory process is in an active stage, and where there has been little or no destruction of nerve tissue. In many such cases the results of treatment are among the most brilliant to be found in the domain of medicine. In cases, however, of long standing where extensive destructive changes have taken place, no treatment will restore dead tissue.

### Symptoms.

There are certain symptoms which should make the physician suspicious of syphilitic disease of the nervous system, no matter what history the patient gives. It should be remembered that an indignant denial of the possibility of having had syphilis, the possession of healthy children, and of previous perfect health in man or woman of any age and any station in life is of no value, and does not constitute sufficient grounds for excluding syphilis. Furthermore it must be remembered that not infrequently extragenital syphilis is encountered especially in physicians, nurses, and others who might be exposed to infection through the hands. In some cases the primary

FIG. I

FIG. II

Figure I. A simple device for taking the pressure of the spinal fluid while it is being withdrawn.

Figure II. Apparatus for the intraspinal injection of salvarsanized serum.

lesion is about the lip or tongue and occasionally in other parts of the body. Frequently, especially in women, syphilis is innocently acquired, and the primary and even secondary manifestations of the disease are not sufficient to excite attention or to awaken suspicion. For these reasons, a negative history is of little or no value, and the physician must be on the qui vive for suspicious symptoms. The most common symptoms are:

1. Any type of convulsion occurring after the age of thirty.
2. Many types of paralyses, occurring before middle life, where high blood pressure and nephritis may be excluded.
3. Persistent headaches, the etiology of which is not clear.
4. Persistent or transitory parasthesias.
5. Many forms of eye disturbances, such as absence of light reflexes in the pupil, rigidity of the pupil, irregularity in the size and shape of the pupil.
6. Various paralyses of the extrinsic muscles of the eye.
7. Ptosis, transitory or permanent, and many disturbances of the optic nerve itself.
8. Any of the classical symptoms of tabes or paresis.

The symptoms enumerated are by no means exhaustive. It might be said by way of summary that any motor or sensory disturbance may rest on a syphilitic base.

Any of the symptoms above indicated, no matter what the history, should always excite suspicion of syphilis. To determine whether or not syphilis is present we must rely on clinical examination and laboratory findings.

### Clinical Examination.

There are certain objective symptoms in syphilis of the nervous system, which are present in a large majority of cases. The symptoms are:

1. Disturbances of the Eye.—On examination the pupils are either sluggish in their reaction to light, or fail entirely to respond to light. The reaction to accommodation may be present or absent. The pupils may be either dilated or contracted, sometimes of normal size. It is extremely common to find an irregularity in the contour of one or both pupils, and a difference in size of the two pupils. If any of these abnormalities are present, it is very suggestive of syphilis.

2. Variations in the Deep Reflexes Particularly the Knee Jerk and the Ankle Jerk.—Exaggeration of the reflexes, or a diminution, or loss, or a difference in response on the two sides associated with any of the symptoms described above are always suggestive.

3. The existence of Romberg's sign is always suspicious.

Many other objective findings are frequently observed, but these just given constitute the most important and should always be looked for. However, even if all are absent, the blood and spinal fluid should in every case be examined.

### Laboratory Examination.

In order to determine definitely whether a clinically suspicious case is syphilitic, a laboratory examination is imperative for accurate diagnosis, and later to determine whether a cure is complete.

The laboratory examination of a patient to determine whether a given nervous disease is of syphilitic origin consists in tests on the blood and cerebrospinal fluid. The Wassermann reaction and provocative Wassermann are of value if they are positive, however, a negative Wassermann on the blood of an individual suffering from a syphilitic disease of the nervous system is of little value, since in these conditions the blood is negative in a great many cases. But an examination of the spinal fluid enables us to tell with absolute certainty as to the syphilitic nature of the disease. Because, here, in addition to the Wassermann reaction we have the cell count which is of no less value. To summarize, we might say that the laboratory examination in these cases consists in the "four reactions" of Nonne, which are:

1. Wassermann Reaction on Blood.—If positive, this shows us we are dealing with a patient who has syphilis whether or not the particular affection which we are trying to diagnose is of syphilitic origin. However, if this is negative it should be disregarded and the spinal fluid examined.

2. Wassermann Reaction on Spinal Fluid.—This should not only be done by the original method of using .2 c. c. of the fluid, but also, with increasing quantities from .3 c. c. up to 1 c. c. of the fluid. It has been shown that it is necessary to use these larger quantities in order to determine if there are present any substances which have the power of fixing complement.

3. Cell Count of the Spinal Fluid.—This should be done on a Fuchs-Rosenthal counting chamber on account of the larger ruled surface and the greater depth of the chamber, thereby insuring greater accuracy in the counts. Quite often there are so few cells present that they can not be correctly estimated by using the ordinary blood counting chamber.

Normally the cell count varies from 0 to 5 or 6 per cu. mm., but when the count goes over 10 it is an indication that we are dealing with a pathological fluid, or in other words, we have an organic disease of the nervous system. This within itself would not enable us to say that the case was syphilis, but this, together with a positive Wassermann reaction would serve to establish the diagnosis beyond a doubt as a syphilitic disease of the nervous system.

4. The Globulin Estimation, "Phase I" of Nonne.—This consists in testing for an increase in the globulins by the use of a hot saturated solution of ammonium sulphate. The spinal fluid is allowed to run down the side of tube having some of the ammonium sulphate solution in the bottom of it. If the globulins are increased a white ring of contact will be formed where the two fluids meet. (The butyric acid test of Noguchi may also be used for the globulin estimation.) This test is found to be positive in a large majority of these cases, however, it may sometimes be negative. We consider it the least important of the four reactions.

ILLUSTRATIVE CASES FROM PRIVATE PRACTICE OF ONE OF US. (DR. GAINES.)

CASE 1.—A man forty-two years of age for six or eight weeks has complained of nocturnal dyspnoea, loss of weight from two hundred and eighty-five to two hundred and thirty-five pounds, nervousness, loss of strength, backache and headaches worse at night, history of chancre fifteen years ago. Physical examination was practically negative, but the Wassermann test on the blood showed a positive reaction (+ + + +). Complete recovery followed antisyphilitic treatment.

CASE 2.—A man 38 years of age who denied any possibility of syphilitic infection, father of five healthy children, and who had always enjoyed good health, two months before being seen, had a convulsion, and during the week prior to being seen, had two more convulsions. Clinical Examination was entirely negative. Wassermann on the spinal fluid, positive, 37 cells to the cubic millimeter in the spinal fluid—an undoubted case of syphilis of the brain, probably of the convexity.

CASE 3.—A woman 32 years of age, who gave a history of chancre, one year ago complained of headaches, weakness, loss of flesh, and double vision. Physical examination revealed papulo-squamous eruption over the body, third nerve paralysis and iritis, with marked tenderness over the skull on percussion (a condition frequently found in syphilis of the brain) Wassermann of the blood positive. This patient fully recovered clinically after anti-syphilitic treatment.

CASE 4.—A man 38 years of age who in 1890 had a chancre on his lip followed by secondaries in the skin, and who took mixed teatment for two years complained of lancinating pains in both legs, occurring about once a months, of two or three years duration, leg weariness, some dribbling of the urine and difficulty in walking in the dark. Physical examination showed Argyll-Robertson pupil, left pupil larger than right, both irregular in contour, absence of knee and ankle reflexes. Romberg signs positive with positive (+ + + +) Wassermann on spinal fluid. Cell count 47 cells per cm. Globulins increased. This is an undoubted case of classical tabes. He was given salvarsanized serum intraspinally, with moderately severe pains in the legs a few hours following. He has been considerably improved as regards pain, sphincter disturbance, and there is less difficulty in walking. This patient is under treatment at the present time, and he will receive further doses at two or three weeks' intervals until the spinal fluid becomes negative. (This patient was kindly referred by Dr. C. W. Strickler, of Atlanta.)

Case 5.—Boy, age 13, whose parents had been treated for syphilis before his birth, four months before being seen, became unconscious during the night, and remained so for nearly twenty-four hours. For some time previous to this seizure, his gait had been noticed to be clumsy, but for the last few months it had been stumbling in character. His speech has also become affected, words being slurred over, and frequently the speech staccato-like.

For the past several years the boy has exhibited marked mental depression with irritability, and for the past few months there has been marked emotional instability, as shown by outbursts of anger, and by silly laughter without adequate cause. A Wassermann on the blood five months before being seen was reported negative. At that time Dr. F. P. Calhoun of Atlanta reported optic neuritis with atrophy. Physical examination showed widely dilated pupils which reacted very sluggishly to light, and nystagmus. The gait was found to be as described in the history, and the boy presented a picture of progressive mental deterioration with motor changes as shown by disturbances in gait and speech. The knee jerks were greatly exaggerated. Spinal fluid Wassermann positive (+ + + +), 87 cells per cu. millimeter, slight excess of globulin. This is a definite case of Juvenile Paresis. When it affects the young it usually manifests itself more frequently in motor disturbances than in the characteristic mental picture.

Mentally such patients frequently show only a gradually deepening dementia. This patient had two intra-spinal treatments of salvarsanized serum with no improvement manifested, either clinically or in the spinal fluid. A recent examination of the eye grounds, however, by Dr. Calhoun showed a marked improvement.

### Treatment.

In no field of medicine are the results of treatment more gratifying than in many of the cases of syphilis of the nervous system. If untreated, or improperly treated, a terrible fate may be considered in store for these unfortunates. Unless prompt and efficacious measures are adopted, prolonged and painful disability and a progressive course may usually be expected. It is important to have a plan of treatment therefore and adhere to it.

First: Intravenous Salvarsan Therapy.—There are two ways in which salvarsan may be administered to these cases, namely, repeated intravenous injections of salvarsan or intraspinal injections of salvarsanized serum. If the simple intravenous method is used, from .3 to .6 gm. should be given at intervals of ten days to two weeks for four to eight doses, and a month later a Wassermann done on the blood, and the spinal fluid examined. If these tests show an absence of active syphilis, and if the clinical symptoms have cleared up, further salvarsan may be deferred. It is wise, however, to have a blood Wassermann and provocative Wassermann done at intervals of three or four months for two or three years. Should the reaction become positive, further salvarsan should be given. Many times it is impossible to obtain a negative Wassermann, even though the clinical symptoms have cleared up. It is our practice to give these cases injections of salvarsan at intervals of two to four weeks, and be guided by clinical manifestations.

If the Wassermann in blood or fluid continues positive the intra-spinal method should be considered.

There is a general belief among students of the subject that a positive Wassermann test means active syphilis, and is an indication that treatment should be continued. It is thus clear that a persistent, positive test indicates the necessity for further treatment, while the finding of a negative reaction does not permit indefinite cessation of treatment. In this connection it is interesting to note that Boas has shown that relapses are preceded by a return of the positive reaction. It is thus important to check up clinical improvement by a frequent Wassermann test of the blood and of the fluid. We consider it important to employ mercury and frequently potassium iodide. In many cases it is advisable to institute a preliminary course of mercury, or a combination of mercuy and iodides before the use of salvarsan. This is done to prevent the appearance of Herxheimer reactions. There is reason to believe that Herxheimer reactions (focal irritation) produced by the "endotoxins," which are set free from the killed spirochaetae in the meninges, may be frequently responsible for alarming symptoms which develop after the use of salvasan, either intravenously or intra-spinally.

Second: Intra-Spinal Injections of Salvarsanized Serum.—This method of applying salvarsan was introduced by Swift and Ellis of the Rockefeller Institute. Since their introduction of this method a number of workers in different parts of the country have reported its use. At the last meeting of the American Medical Association at Atlantic City, it was stated by those who have had the most experience with this method that it is a valuable procedure in all forms of nervous syphilis, except in paresis. It may be said that no case of paresis has yet been cured. The advantage of the method is to secure more rapidly the application of the salvarsan to the seat of the active syphilitic inflammatory process in the brain or cord.

It appears that in certain types of syphilitic nervous diseases salvarsan given intravenously penetrates to the recesses of the brain and cord very slowly. The method of giving this treatment which we have used with success is as follows:

The patient is given an ordinary dose of salvarsan intravenously. One-half to one hour later fifty to sixty c. c. of blood is withdrawn from a vein directly into centrifuge tubes. We draw the blood directly into centrifuge tubes so as to minimize the handling and thus lessen the danger of infection and also insure a perfectly clear hemoglobin-free serum. After the serum has been separated it is diluted to 40% with freshly distilled normal salt solution. This is then heated to 56° C. for twenty minutes so as to inactivate it. In the adult we usually begin with 12 c. c. of serum diluted to 30 c. c. with salt solution. If this is tolerated well at the next injection we use a 50% serum and in some cases, where it seems to be indicated, we increase the strength at each injection until the pure serum is used. With reference to the amount and strength of the serum used we are guided largely by the conditions and resistance of each individual patient.

The exact method of introducing the serum is as follows: A lumbar puncture with the patient in the recumbent position is made on the day following the injection of salvarsan and from twenty to forty c. c. of spinal fluid is withdrawn drop by drop. The apparatus shown in Figure II is used for the injection by the gravity method. We think the gravity method superior to that of using a syringe as a more steady and even flow can be obtained, and besides, by using this method there is no danger of exerting too much pressure. The rubber tubing shown is forty cm. long with a metal connection which fits snugly into the lumbar puncture needle. After the needle has been inserted and the fluid withdrawn, (the prepared serum having been previously put into the container, which has been sterilized, shown in Figure II) the tubing is connected to the needle and the serum is allowed to flow very slowly and gradually into the spinal canal. Care should be taken that no air bubbles are introduced and that too much pressure is not exerted by raising the container too high suddenly. After the serum has been introduced the needle is withdrawn and the puncture sealed with collodion. Of course, the whole procedure should be done under the strictest aseptic precautions. The patient is then kept in bed for twenty-four hours.

Frequently there is some pain following the injection, more frequently in the legs. For the relief of pain MORPHINE should never be used as serious symptoms have followed its administration, probably from its effects upon the respiratory center. The best sedatives to use under these conditions are phenacetin and codeine. The pain usually disappears after a few hours.

### Conclusions.

Syphilis of the nervous system is a frequent disease, far more frequent than was formerly believed. Many cases diagnosed rheumatism, auto-toxemia, malaria, eye-strain, migraine, chronic Bright's disease, epilepsy, hysteria and numerous other maladies turn out to be cases of syphilitic disease, and frequently are entirely relieved by energetic and systematic treatment.

When a destructive process has occurred, improvement is questionable, but further progress of the disease, we confidently believe, may be checked. Where there is little or no destruction of nerve tissue, we may expect complete functional restoration.

In the pre-ataxic stage of tabes, the results of the intra-spinal injection of salvarsanized serum are often brilliant. In the ataxic stage, there is frequently a great improvement in walking, especially if re-education methods are employed.

In the paralytic stage, little may be expected. In all stages, lancinating pains are usually relieved.

The effect of treatment should be checked up, not only by clinical observation, but by examination of the spinal fluid. Treatment should be persisted in until the fluid is normal.

If the intra-spinal treatment is given according to the technic described, there is practically no danger.

When one remembers that syphilis of the nervous system in many cases, and particularly in tabes, has been considered a progressive, incurable disease, it is certainly worth every effort to give these patients the advantage of the most successful form of treatment that has yet been devised.

1023 Empire Building.
214 The Grand.

## Figures and Tables

**FIG. I. FIG. II f1:**